# Duplex ultrasound and computed tomography angiography in the follow-up of endovascular abdominal aortic aneurysm repair: a comparative study*

**DOI:** 10.1590/0100-3984.2014.0139

**Published:** 2016

**Authors:** Alex Aparecido Cantador, Daniel Emílio Dalledone Siqueira, Octavio Barcellos Jacobsen, Jamal Baracat, Ines Minniti Rodrigues Pereira, Fábio Hüsemann Menezes, Ana Terezinha Guillaumon

**Affiliations:** 1 Vascular Surgeon, Private practice/consultancy, Campinas, SP, Brazil.; 2 Graduate Student in the Department of Vascular Diseases, Faculdade de Ciências Médicas da Universidade Estadual de Campinas (FCM-Unicamp), Campinas, SP, Brazil.; 3 PhD, Assistant Professor in the Department of Radiology, Faculdade de Ciências Médicas da Universidade Estadual de Campinas (FCM-Unicamp), Campinas, SP, Brazil.; 4 PhD, Assistant Professor in the Department of Vascular Diseases, Faculdade de Ciências Médicas da Universidade Estadual de Campinas (FCM-Unicamp), Campinas, SP, Brazil.; 5 PhD, Associate Professor, Head of the Department of Vascular Diseases, Faculdade de Ciências Médicas da Universidade Estadual de Campinas (FCM-Unicamp), Campinas, SP, Brazil.

**Keywords:** Aneurysm, Aorta, abdominal, Endovascular procedures, Ultrasonography, Angiography/methods

## Abstract

**Objective:**

To compare duplex ultrasound and computed tomography (CT) angiography in terms of their performance in detecting endoleaks, as well as in determining the diameter of the aneurysm sac, in the postoperative follow-up of endovascular abdominal aortic aneurysm repair.

**Materials and Methods:**

This was a prospective study involving 30 patients who had undergone endovascular repair of infrarenal aortoiliac aneurysms. Duplex ultrasound and CT angiography were performed simultaneously by independent radiologists. Measurements of the aneurysm sac diameter were assessed, and the presence or absence of endoleaks was determined.

**Results:**

The average diameter of the aneurysm sac, as determined by duplex ultrasound and CT angiography was 6.09 ± 1.95 and 6.27 ± 2.16 cm, respectively. Pearson's correlation coefficient showing a statistically significant correlation (*R* = 0.88; *p* < 0.01). Comparing the duplex ultrasound and CT angiography results regarding the detection of endoleaks, we found that the former had a negative predictive value of 92.59% and a specificity of 96.15%.

**Conclusion:**

Our results show that there is little variation between the two methods evaluated, and that the choice between the two would have no significant effect on clinical management. Duplex ultrasound could replace CT angiography in the postoperative follow-up of endovascular aneurysm repair of the infrarenal aorta, because it is a low-cost procedure without the potential clinical complications related to the use of iodinated contrast and exposure to radiation.

## Abstract (pt)

**Objetivo:**

Comparar a ultrassonografia duplex e a angiotomografia computadorizada no diagnóstico dos *endoleaks* e na medida do diâmetro do saco aneurismático no acompanhamento pós-operatório da correção endovascular do aneurisma de aorta abdominal.

**Materiais e Métodos:**

Foram estudados, prospectivamente, 30 doentes submetidos a correção endovascular de aneurismas de aorta infrarrenal e ilíacas. No seguimento pós-operatório foram realizadas ultrassonografia duplex e angiotomografia no mesmo intervalo de tempo, por radiologistas independentes. Foram avaliadas as medidas do saco aneurismático e a presença ou ausência de *endoleaks*.

**Resultados:**

O diâmetro médio do saco aneurismático encontrado foi 6,09 ± 1,95 cm para a ultrassonografia duplex e 6,27 ± 2,16 cm para a angiotomografia, existindo correlação estatisticamente significante, com *R* = 0,88 e *p* < 0,01 (Pearson). Considerando a detecção de *endoleaks*, o valor preditivo negativo da ultrassonografia duplex (comparada à angiotomografia) foi 92,59% e a especificidade foi 96,15%.

**Conclusão:**

Os resultados demonstram pequena variação entre os métodos empregados, não comprometendo o manejo clínico. A ultrassonografia duplex poderia substituir a angiotomografia no acompanhamento pós-operatório da correção endovascular do aneurisma de aorta infrarrenal, com baixo custo, evitando potenciais complicações clínicas relacionadas ao uso de contraste iodado e exposição à radiação ionizante.

## INTRODUCTION

There has been rapid growth in the endovascular treatment of abdominal aortic aneurysm (AAA), which has become increasingly common in daily practice. Endovascular treatment is a less invasive option, offering patients quicker recovery, as well as less perioperative morbidity and mortality, although it requires closer, lifelong surveillance and more frequent use of ancillary tests, in order to evaluate postoperative complications^(1)^. The purpose of endovascular repair of an infrarenal AAA is to exclude the aneurysm sac, thus avoiding blood flow within the aneurysmal dilatation. Possible postoperative complications include endoleaks (the most frequent complication), continued growth of the aneurysm sac, stent migration, structural failure of the stent, and impaired flow to the lower member due to stenosis or occlusion of an stent branch^(1)^.

Using duplex ultrasound in the postoperative evaluation of patients with infrarenal abdominal aortic stent has been validated in many aspects, including the identification of endoleaks and of alterations in the size of the aneurysm sac^(1-5)^. When compared with computed tomography (CT) angiography, ultrasound has shown a sensitivity of 12-100% and a specificity of 74-99%^(2)^. In a paper published in 2009, Manning et al. showed that ultrasound had a sensitivity of 86% and a negative predictive value of 94%^(2)^ These differences emphasize the examiner-dependent nature of ultrasound, which makes it necessary for each institution to validate their results separately.

When ultrasound is used in order to monitor abdominal stent complications, its application is even more efficient. Endoleaks that require an additional intervention lead to an expansion of the aneurysm sac^(4)^, a characteristic which can be evaluated with ultrasound. Occlusion and stenosis of stent branches, with hemodynamic impairment of the member (revealed by ischemia or claudication), can also be visualized^(1,4)^. Chaer et al. suggested that the fear of catastrophic events, such as rupture, should not affect the choice of surveillance technique, given that such events typically occur after a period of nonadherence to postoperative treatment or after relapse, regardless of the ancillary technique chosen^(1)^.

In the present study we evaluate the postoperative (follow-up) findings of endovascular repair of AAA, using duplex ultrasound, and comparing it with the gold standard method, CT angiography. The objective was to evaluate the reach and efficiency of the method, as well as the possibility of incorporating it into the postoperative follow-up routine for this type of intervention.

## MATERIALS AND METHODS

This was a prospective study involving 30 patients. The study was approved by the Research Ethics Committee of the Universidade Estadual de Campinas (Protocol no. CEP 941/2009), and all of the patients gave written informed consent. The patients were then evaluated, and a detailed report of their risk factors was prepared. There was a predominance of males, who accounted for 83% of the sample. The risk factors observed were smoking (80%), arterial hypertension (73%), diabetes mellitus (30%), dyslipidemia (23%) and myocardial infarction (16%).

At the time of the intervention, the mean age of the patients was 75 years (range, 58-85 years) and the mean diameter of the aneurysm sac was 6.5 cm (range, 3.5-8.8 cm). The time from surgery to postoperative evaluation was different for each patient, the mean time being 12.9 months (range, 2-52 months). We evaluated one ultrasound and one CT angiography per patient, with a maximum interval of two weeks between examinations. Patients who were allergic to iodinated contrast were excluded, as were those with a creatinine level > 2.0 mg/dL. Ultrasound images were obtained after bowel preparation-8 hours of fasting and 40 drops of dimethicone every 8 hours on the eve of the examination. We selected one radiologist to perform all ultrasounds and another to evaluate all CT angiographies. Both were experienced radiologists, certified by the Brazilian College of Radiology and Diagnostic Imaging. The reports were generated independently, without data sharing between the examiners.

**Figure 1:**
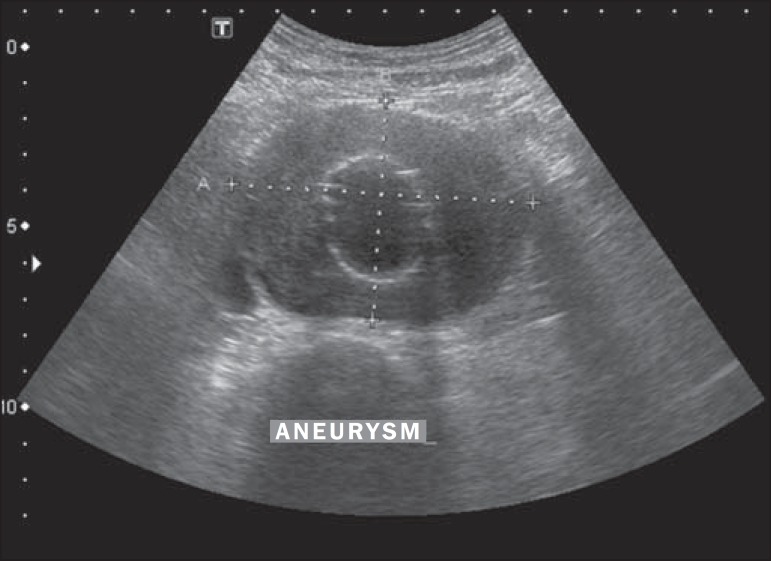
Cross-sectional B-mode ultrasound image showing the measuring of the aneurysm sac. Note the stent within the sac.

**Figure 2:**
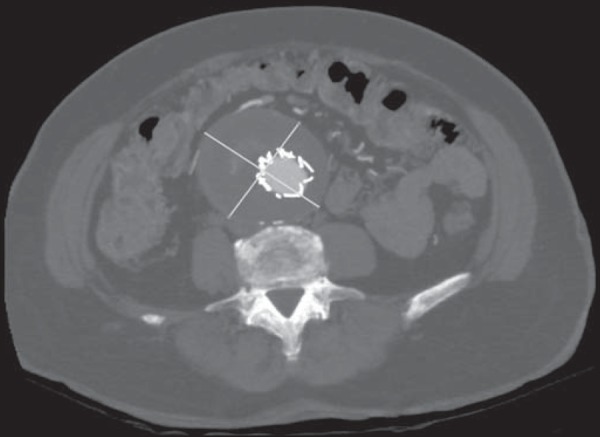
Axial section CT angiography showing the measuring of the aneurysm sac. Note the stent within the sac.

The criteria evaluated were the diameter of the aneurysm sac (Figures 1 and 2) and the presence or absence of endoleaks. In the ultrasound examination, which was performed in B-mode, the diameter was measured along the anteroposterior and laterolateral axes. The measurements obtained by CT angiography were also taken along the anteroposterior and laterolateral axes, in axial sections, without reformatting.

Endoleaks were initially evaluated through the acquisition of good-quality B-mode ultrasound images, the aneurysm sac and stent being inspected in cross-sectional and longitudinal views. Thereafter, we used color Doppler ultrasound, also in cross-sectional and longitudinal views, in an attempt to identify any flow between the stent and the aneurysm sac, taking care to use the appropriate gain adjustment. Finally, endoleaks were evaluated with spectral Doppler ultrasound, in order to confirm the findings of the color Doppler ultrasound examination.

## RESULTS

**Figure 3:**
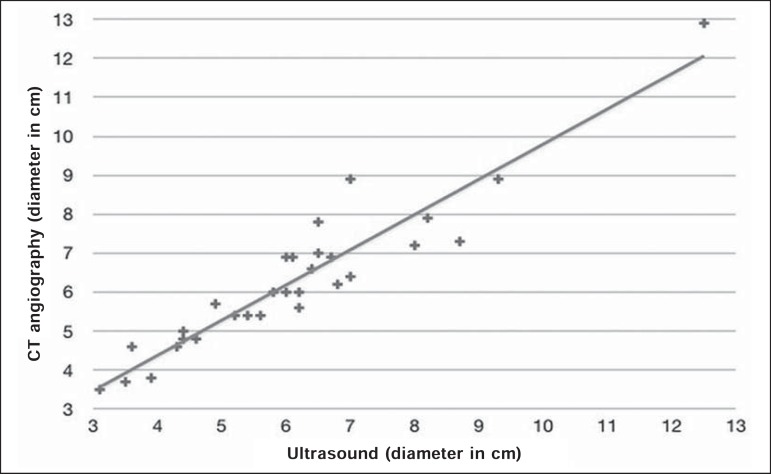
Scatter plot showing the aneurysm sac diameters determined by the two methods under study.

**Figure 4:**
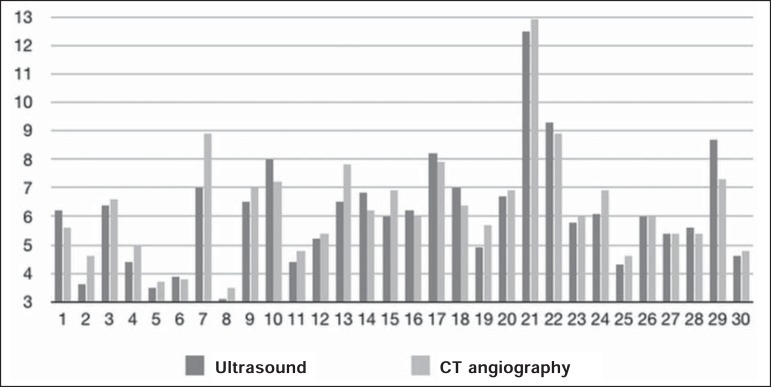
Bar chart showing aneurysm sac diameters determined with the two methods, by patient.

**Figure 5:**
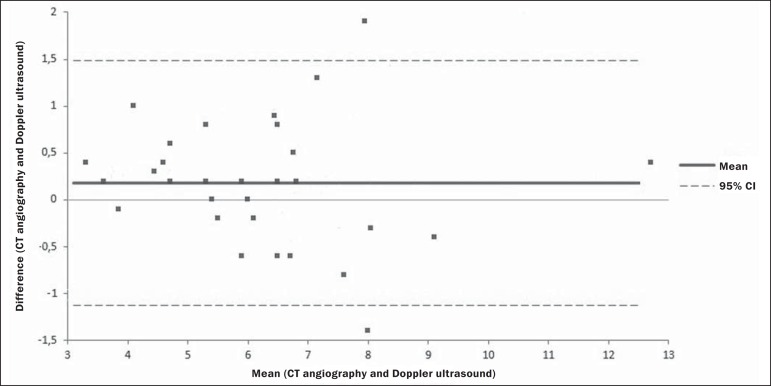
Bland-Altman plot. The mean aneurysm sac diameter, between the methods employed, is represented on the X axis, and the difference between the methods is represented on the Y axis. Each point in the graph represents a patient. The overall mean is indicated by the continuous line, and the confidence interval is indicated by the dashed line.

The mean diameter of the aneurysm sac was 6.09 ± 1.95 cm when determined by duplex ultrasound and 6.27 ± 2.16 cm when determined by CT angiography. Pearson's correlation coefficient revealed a statistically significant correlation (*R* = 0.88; *p* < 0.01), as depicted in Figures 3 and 4. The results are also shown in a Bland-Altman plot (Figure 5).

A total of 4 endoleaks were diagnosed with CT angiography, and the ultrasound examination detected only 2 of those 4. Compared with CT angiography, ultrasound showed a sensitivity of 50% (95% confidence interval [95% CI]: 15-85%), a specificity of 96.15% (95% CI: 81-99%), a positive predictive value of 66.67% (95% CI: 20-93%), a negative predictive value of 92.59% (95% CI: 76-97%), and a kappa value of 0.5161 (95% CI: 0.163-0.869).

## DISCUSSION

The use of CT with intravenous injection of contrast is currently considered the gold standard for regular long-term follow-up of endovascular repair of AAA^(1,3,6)^. However, that is a high-cost examination that exposes patients to ionizing radiation and the risk of renal function impairment and allergy due to the use of iodinated contrast^(1,2,6-8)^. Duplex ultrasound, in addition to detecting postoperative complications, has the advantages of being noninvasive, safer, less expensive, and widely available^(1,3,5)^.

In the present study, we have demonstrated a statistically significant correlation between duplex ultrasound and CT angiography (the gold standard) in terms of their accuracy in determining the diameter of the aneurysm sac.

For the diagnosis of endoleaks, we found that duplex ultrasound showed a specificity of 96.15% and a sensitivity of 50%. The small size of the sample (30 patients) limited the evaluation of sensitivity and specificity. If we consider the formula proposed by Kish^(9)^:

N=Z*Z(P(1–P))/(D*D)

where *N* is the minimum sample size, Z is the area under the normal curve corresponding to the 95% CI, P is the prevalence of the event of interest, and D is the desired precision-attributing an estimated sensitivity of 74% (with a 12% margin of error) and an estimated specificity of 94% (with a 4% margin of error), as in the systematic review recently conducted by Karthikesalingam et al.^(10)^, the minimum required sample size, with a 95% CI, would be 52 patients to calculate sensitivity and 136 patients to calculate specificity.

The endoleaks that were not diagnosed with ultrasound (2 out of a total of 4) were type II endoleaks that showed no expansion of the aneurysm sac and were controlled without surgical intervention. Therefore, despite the sensitivity of 50%, follow-up with ultrasound did not affect the clinical management of the cases.

**Figure 6:**
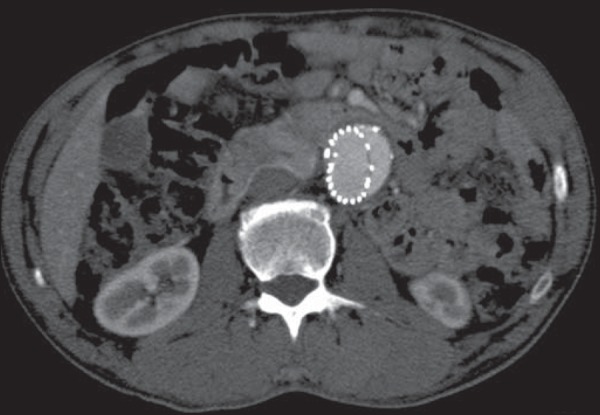
Axial section CT angiography image showing a stent in the infrarenal aorta with a proximal (type IA) endoleak.

According to the systematic review conducted by Karthikesalingam et al.^(10)^, duplex ultrasound has shown high sensitivity and specificity in the diagnosis of type I and III endoleaks, when compared with contrast-enhanced ultrasound and CT angiography, with sufficient accuracy for postoperative monitoring of endovascular repair of AAA. Some of the studies included in that review suggested that the sensitivity of duplex ultrasound would be insufficient to justify its use as the sole follow-up method. However, when the authors considered only the endoleaks classified as type I (Figure 6) or type III, both of which require surgical treatment, the sensitivity showed a significant increase, underscoring the safety of the method.

The study conducted by França et al.^(11)^ showed that ultrasound had a sensitivity and specificity of 54.5% and 92.8%, respectively, for the detection of endoleaks, when compared with CT angiography in a sample of 50 examinations, as well as showing a statistically significant correlation for the measurement of the aneurysm sac (*R* = 0.97; *p* < 0.001). Moraes Filho et al.^(12)^ found that ultrasound had a sensitivity of 75% and a specificity of 96% for the detection of endoleaks and that there was a statistically significant correlation for the aneurysm sac diameter (*R* = 0.91).

It should be borne in mind that obesity and inadequate bowel preparation are limiting factors for abdominal ultrasound examination, due to an inappropriate acoustic window. Other limitations include examiner-dependence and physical variations of patients^(12,13)^. Nevertheless, ultrasound is a reproducible, less expensive, more widely available method than is CT angiography and does not involve the use of iodinated contrast or radiation. Therefore, the routine use of ultrasound could reduce the number of CT angiographies.

## CONCLUSION

Our results indicate that there is a good correlation between the two methods for the evaluation of the aneurysm sac diameter and a reasonable correlation of sensitivity and specificity for the detection of endoleaks, the choice between the two therefore having no affect on clinical management. Duplex ultrasound could complement CT angiography in the postoperative follow-up of endovascular repair of abdominal aortoiliac aneurysms, reducing the potential for clinical complications related to the use of iodinated contrast and exposure to ionizing radiation, in accordance with the studies carried out at other centers.
